# The Influence of Ziegler-Natta and Metallocene Catalysts on Polyolefin Structure, Properties, and Processing Ability

**DOI:** 10.3390/ma7075069

**Published:** 2014-07-09

**Authors:** Ahmad Shamiri, Mohammed H. Chakrabarti, Shah Jahan, Mohd Azlan Hussain, Walter Kaminsky, Purushothaman V. Aravind, Wageeh A. Yehye

**Affiliations:** 1 Department of Chemical Engineering, Faculty of Engineering, University of Malaya, 50603 Kuala Lumpur, Malaysia; E-Mails: a.shamiri@um.edu.my (A.S.); jakirkhanbd@gmail.com (S.J.); mohd_azlan@um.edu.my (M.A.H.); 2 Energy Futures Lab, Electrical Engineering Building, Imperial College London, South Kensington, London SW7 2AZ, UK; 3 Institute for Technical, Macromolecular Chemistry, University of Hamburg, Bundesstr. 45, D-20146 Hamburg, Germany; E-Mail: kaminsky@chemie.uni-hamburg.de; 4 Process and Energy Department, Delft University of Technology, Leeghwaterstraat 44, 2628 CA Delft, The Netherlands; E-Mail: P.V.Aravind@tudelft.nl; 5 Nanotechnology and Catalysis Research Center (NANOCEN), University of Malaya, 50603 Kuala Lumpur, Malaysia; E-Mail: wdabdoub@um.edu.my

**Keywords:** polyolefin, Ziegler-Natta catalyst, methylaluminoxane, metallocene, co-catalysts

## Abstract

50 years ago, Karl Ziegler and Giulio Natta were awarded the Nobel Prize for their discovery of the catalytic polymerization of ethylene and propylene using titanium compounds and aluminum-alkyls as co-catalysts. Polyolefins have grown to become one of the biggest of all produced polymers. New metallocene/methylaluminoxane (MAO) catalysts open the possibility to synthesize polymers with highly defined microstructure, tacticity, and steroregularity, as well as long-chain branched, or blocky copolymers with excellent properties. This improvement in polymerization is possible due to the single active sites available on the metallocene catalysts in contrast to their traditional counterparts. Moreover, these catalysts, half titanocenes/MAO, zirconocenes, and other single site catalysts can control various important parameters, such as co-monomer distribution, molecular weight, molecular weight distribution, molecular architecture, stereo-specificity, degree of linearity, and branching of the polymer. However, in most cases research in this area has reduced academia as olefin polymerization has seen significant advancements in the industries. Therefore, this paper aims to further motivate interest in polyolefin research in academia by highlighting promising and open areas for the future.

## 1. Introduction

**Figure 1:**
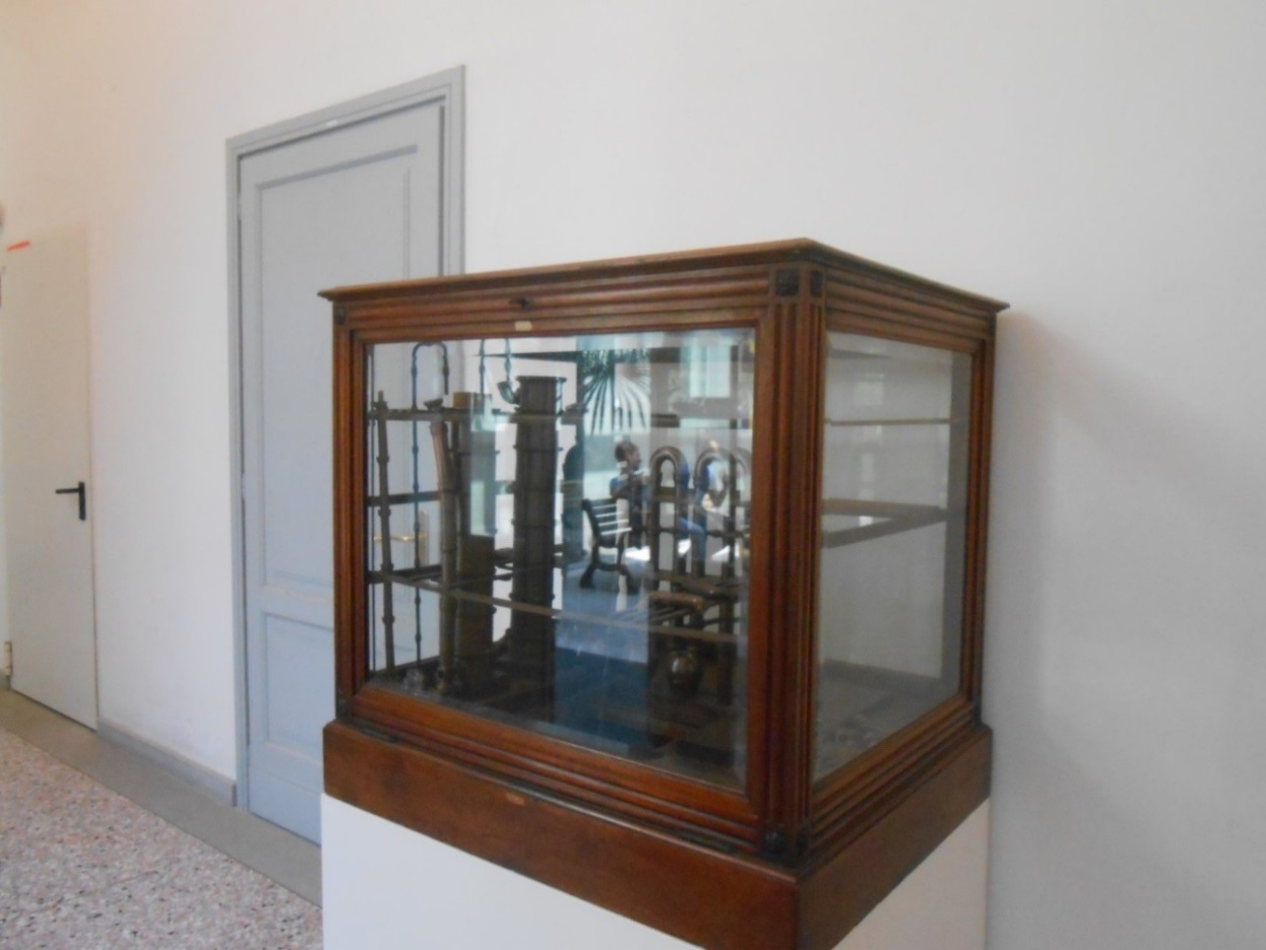
The original equipment used by Karl Ziegler for discovering his catalyst and co-catalyst systems for the polymerization of ethylene to high-density polyethylene (HDPE).

One of the most important discoveries in chemistry and in the chemical industries in the last century is that of the Ziegler-Natta catalysts for the polymerization of olefins [1,2,3]. A catalyst is used to reduce the activation energy for the polymerization process thereby speeding up the reaction and allowing it to proceed even under mild conditions. In 1953, Karl Ziegler discovered the catalyst based on titanium tetrachloride (TiCl_4_) and diethylaluminium chloride [(C_2_H_5_)_2_AlCl] as a co-catalyst for the polymerization of ethylene [4,5] into high molecular weight HDPE (high density polyethylene) at room temperature (Figure 1 shows a photo of the original equipment employed by Ziegler) [3,4,5,6,7]. Furthermore, this catalyst was utilized by Giulio Natta to polymerize propylene into crystalline PP (polypropylene) [8]. Karl Ziegler and Giulio Natta became Nobel Laureates 50 years ago, in 1963, for their respective discoveries in the field of polymers [9]. The discovery of Ziegler-Natta catalysts gave a new dimension to the world of polymers. For more than five decades remarkable progress in catalytic olefin polymerization simplified polyolefin production by eliminating deactivation, solvents, and polymer-purification steps. It seemed that catalyst design, polymer reaction engineering, and polymer process technologies were being pushed forward to produce novel polyolefin materials to meet the demands of highly diversified industries [4,10].

Ziegler-Natta catalysts are the most popular ones employed within the global polymerization industry for the production of PP [11,12]. On the basis of solubility, the Ziegler-Natta catalyst has been categorized into two major classes:

(i) Heterogeneous catalysts: These are industry-dominating catalysts that are based on titanium compounds (and sometimes vanadium-based) and used for polymerization reactions, usually in combination with organo-aluminum compounds like tri-ethylaluminium (TEA=Al(C_2_H_5_)_3_) as co-catalysts [3,13].
(ii) Homogeneous catalysts: These are the second broad class of catalysts and are based on complexes of Ti, Zr, or Hf. They are generally used in combination with a range of different organo-aluminum co-catalysts known as metallocene/methylaluminoxane (MAO). Traditionally, they include metallocenes but also feature multi-dentate oxygen- and nitrogen-based ligands [14,15].

Heterogeneous Ziegler-Natta catalysts are composed of titanium tetrachloride which is supported on magnesium chloride by means of tri-ethylaluminium (AlEt_3_) or AlEt_2_Cl as co-catalysts [5,8,16]. To improve the stereo control of the propylene polymerization process, Lewis bases such as ethyl benzoate, silanes, or other donors are added [3]. Since heterogeneous catalysts are complex systems with different active sites, the polymer structure is influenced only to a limited extent.

In the early 1970s, new catalysts containing magnesium compounds (such as magnesium chloride or magnesium alkoxide, in conjunction with either TiCl_4_ or TiCl_3_) were designed that improved the activity of Ziegler-Natta catalysts and trialkylaluminium co-catalysts by at least one or two orders of magnitude [3]. Catalyst efficiencies of 100 to 1000 kg polymer per gram of titanium were reported. These magnesium/titanium-based catalysts were designated as second-generation Ziegler-Natta catalysts. Due to their very high activities, the residual catalysts did not need to be removed from the polymers and, consequently, catalyst removal steps were no longer necessary as part of the manufacturing process.

**Figure 2:**
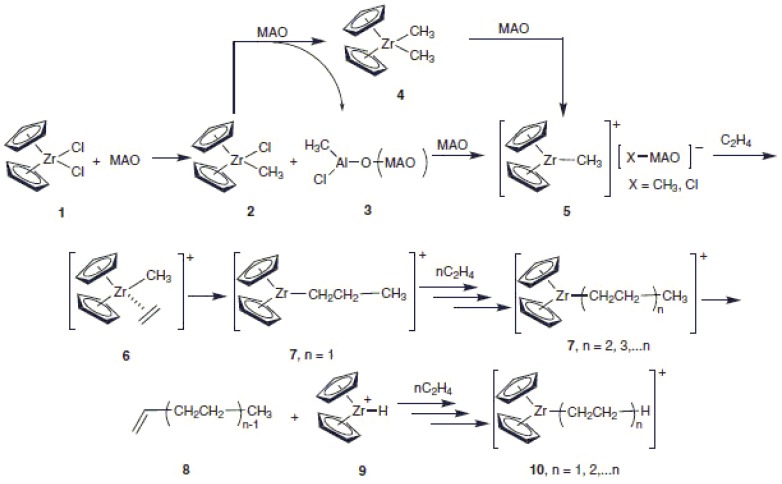
Proposed mechanism of Ziegler-Natta polymerization of C_2_H_4_ using a homogenous catalyst ZrCp_2_Cl_2_/MAO (Cp = cyclopentadienyl; Zr = zirconium; MAO = methylalumoxane), reprinted with permission from [17], copyright 2011 the Brazilian Chemical Society.

Figure 2 represents the mechanism for the catalysis of polyolefins [17]. The treatment of a toluene solution and zirconocene dichloride (or ZrCp_2_Cl_2_) (**1**) with MAO (methylaluminoxane) results in a rapid initial ligand exchange reaction that firstly generates the mono-methyl complex Cp_2_ZrCH_3_Cl (**2**). Note that Cp_2_ refers to cyclopentadienyl. Based on solid-state XPS and ^13^C-NMR studies, as well as investigations on Cp_2_Zr(CH_3_)_2_/MAO solutions, researchers show that an excess of MAO leads to the generation of Cp_2_ZrMe_2_ (**4**), and the catalytically active ion-paired species [Cp_2_ZrCH_3_]^+^ (**5**) along with the counter ion [X-Al(Me)O^−^]*_n_*^−^ (X = Cl, Me) [3]. The cation Cp_2_ZrCH_3_^+^ (**5**) in the presence of ethylene results in a π-complex (**6**) that in turn gives the insertion product (**7**) (*n* = 1) as the first intermediate of the polymerization process. This is followed by a step-by-step insertion of ethylene achieving the cationic alkyl zirconocene (**7**) (*n* = 2, 3... *n*). β-Elimination gives the uneven chain polymer containing a terminal C=C double bond (**8**). The cationic zirconocene hydride (**9**) commences the polymerization reaction that is catalyzed by a zirconocene cation to give an even chain polymer (**10**). For further details, the reader is referred to the publication by Santos [17].

**Figure 3:**
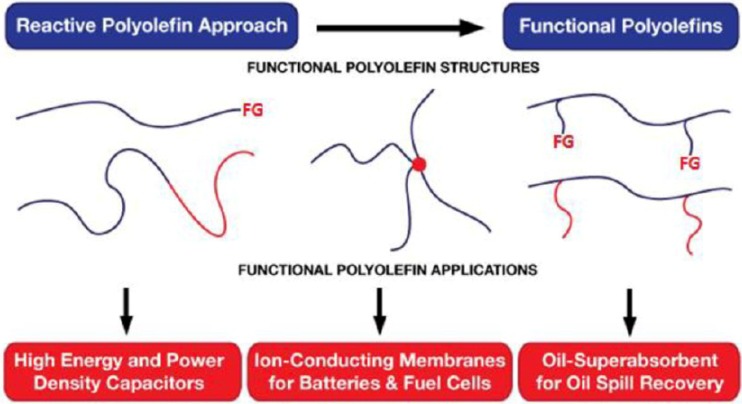
Functional polyolefins for energy applications. Adapted with permission from [2]. Copyright 2013 ACS.

The upsurge in the interest for the synthesis of polyolefins is due to their versatile applications from daily life to high performance engineering applications as represented in Figure 3 [2]. PP (polypropylene) is similar to PE (polyethylene) but has the methyl group (–CH_3_) attached to alternate carbon atoms of the chain. PP’s molecular weight typically lies within 50,000 to 200,000 g·mol^−1^. Table 1 provides some of the physical properties of PE and PP [3].

**Table 1:** Physical properties of polyethylene (PE) and polypropylene (PP), reprinted with permission from [3]. Copyright 2013 World Scientific.

No.	Properties	Polyethylene	Polypropylene
1	Density	0.92–0.95	0.9–0.91
2	Young Modulus (GPa)	0.3–1.0	1.4
3	Glass Transition Temperature (°C)	−125–−80	−20
4	Limiting oxygen index (LOI) (%)	18	17
5	Melting temperature (°C)	112–134	160
6	Specific Heat Capacity: Conventional (J/kg·K)	1750–2400	1900
7	Specific Heat Capacity: Volumetric (10 J/m·K)	1600–2200	1700
8	Speed of sound (10 m/s)	18–32	34–39
9	Stiffness to weight ratio: Tensile (MN-m/kg)	0.32–1.0	1.2–1.5
10	Stiffness to weight ratio: Tensile, Ultimate (KN-m/kg)	7.6–52	25–39
11	Tensile Strength: Ultimate (MPa)	7–49	23–36
12	Thermal Conductivity Ambient (W/m·K)	0.36–0.45	0.15

**Figure 4:**
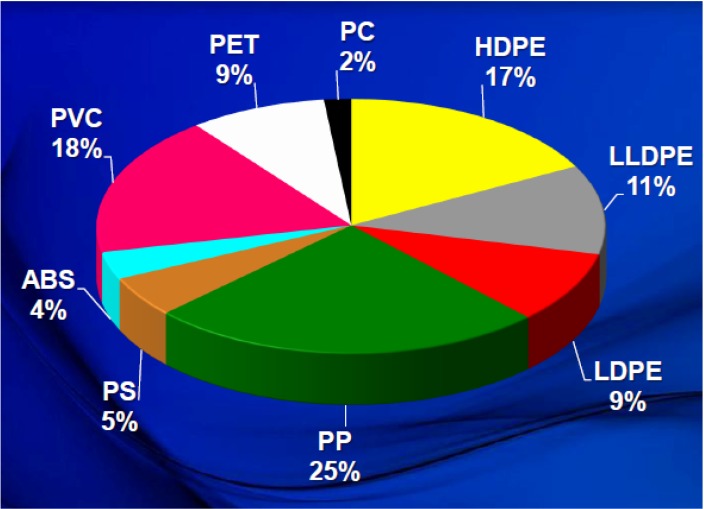
A pie chart showing that polyethylene is the most widely used polymer worldwide (the 2012 total for world polymer demand is 211 million metric tons), reprinted with permission from [18]. Copyright 2012 IHS Inc.

The world’s consumption of low-density polyethylene (LDPE), high-density polyethylene (HDPE), linear low-density polyethylene (LLDPE), and PP was greater than 100 million tons in 2006 and reached a total of 131 million metric tons in 2012 (37% of PE and 25% of PP, as shown in Figure 4) [18]. This significant increase occurred due to the polyolefins inherent properties and wide range of applications. Such polyolefins could be recycled mechanically or by incineration that did not result in any toxic discharges. However, their incapability of decomposing under natural conditions caused a great deal of environmental concern for their packaging uses. Since they constitute a considerable percentage of domestic garbage, polyolefins tend to fill up landfill sites by a significant amount. Therefore, some research activities are focusing on sustainable polyolefin production that can save on energy and raw material consumption for future generations [19,20,21].

This paper provides an overview of the catalytic polymerization of both ethylene and propylene. This is followed by a detailed discussion of the catalyst and co-catalyst systems employed for polymerization (commencing with Ziegler-Natta catalysts and leading to zirconocene, MAO, and titanocene co-catalyst systems). Unfortunately, the amount of publications in this field is declining because sufficient research infrastructure is not present in academic research institutes. Greater research is being conducted by industries and this paper aims to restore the interest in polyolefins within academic institutions.

## 2. Polyethylene and Polypropylene

PE is the most popular and widely used polymer to date [22]. The formation of PE occurs by the polymerization of the ethylene monomer in an insertion reaction. Despite the simple structure of PE, its manufacturing route is quite complex with different types of synthetic procedures [3]. Due to some of its peculiarities, it is considered as a unique polymer having high crystallization rate and chain flexibility, which are mostly derived from its perfect chain structure [23]. Therefore, it is not available in an amorphous state and most of its properties are derived by extrapolating from those of semi crystalline samples. The properties of different forms of PE can vary as a consequence of structural changes resulting from the polymerization technique.

**Figure 5:**
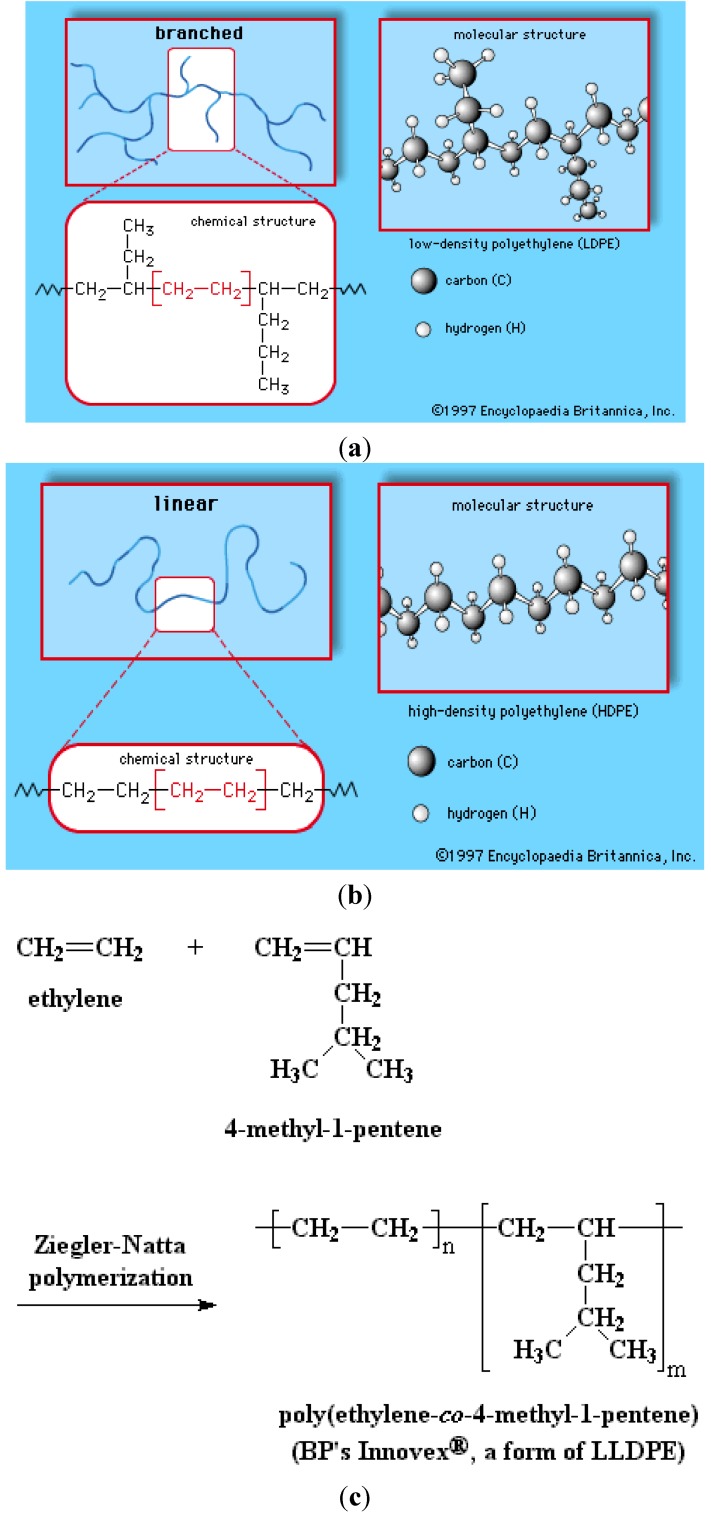
General representations of various polyethylene variants. (**a**) LDPE; (**b**) HDPE; and (**c**) LLDPE. Obtained with permission from MAG Recycling Services Pty Ltd. [30] and the University of Southern Mississippi [31].

In general, LLDPE and HDPE are conventionally synthesized via the catalytic ethylene polymerization reaction at low temperatures and pressures, as compared to the LDPE manufacturing route [24]. In particular, LLDPEs prepared via Ziegler-Natta catalysis have more uneven co-monomer distributions, whereas, a reverse trend is observed for those synthesized by metallocene catalysts. Such differences in co-monomer distributions are mainly attributed to the difference in the available active sites in the two catalysts that manifests itself in the rheological and mechanical properties of the polymers as well as their melt miscibility. However, polymer density can be controlled by the ethylene/co-monomer molar ratio, temperature, and the catalyst type. The ability to crystallize the substance is affected by its molecular weight, concentration of branches, and their distribution along the backbone of the co-polymer [25]. In order to understand the crystallization behavior of the branched molecules, more homogeneous fractions of the co-polymer are required [3]. The processing ability and the properties of the final product depend strongly on the branching of the polymer. The microstructure of the three classes of PE is shown schematically in Figure 5 [3].

The macroscopic properties of polyolefins strongly depend on the chain structure and therefore, the quality of PE in both molten and solid state could be tuned by the presence of side chains of various lengths and quantities [26]. This dependence is caused by steric hindrances of the side chains that affect primarily the polymer’s crystalline nature [15,27]. Generally agreed models also suppose that the side chains are incorporated in the amorphous phase and only a small portion of the side-chain atoms are located inside crystalline regions, where they create packing errors [28]. Kaminsky and co-workers also suggested that in some cases these short chains, namely those based on rather long co-monomers, can crystallize and possibly create separated aggregates [29].

A large fraction of HDPE is produced using catalysts developed by the Phillips Petroleum Company, which are CrO_3_ supported on SiO_2_–Al_2_O_3_. These and other supported transition metal oxide catalysts were discovered in Phillips’ and Standard Oil’s laboratories respectively at about the same time as the Ziegler catalyst. Apart from HDPE, various LLDPEs are commercially produced using the supported Ziegler catalysts. These catalysts account for about 90% of the world’s production while the rest of the 10% are handled by metallocene catalysts [32,33,34,35].

This leads to a brief discussion on mono-modal and multimodal PEs as these have significant differences in their properties [3]. Multimodal means that two or more peak molecular weights can be seen by gel permeation chromatography (GPC). For example, a bimodal PE means that two peak molecular weights can be identified. Multimodal PE can be transformed into articles by injection molding, blow molding, rotational molding, and film extrusion. One of the advantages of multimodal PE over mono-modal PE is its easier and faster processing with reduced energy requirement and increased output. In addition, multimodal PEs show less flow disturbances in thermal processing.

**Figure 6:**
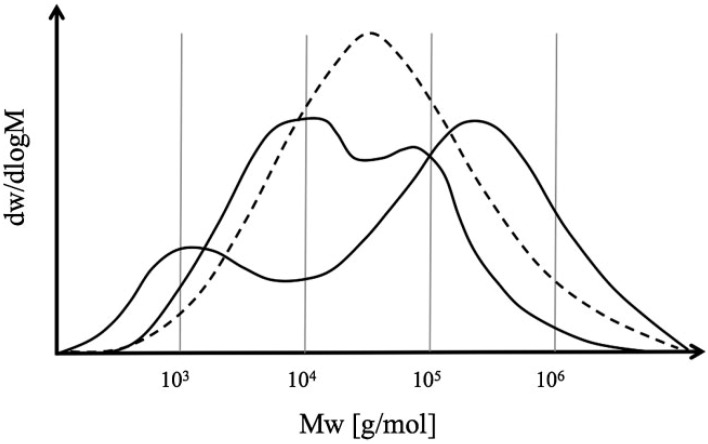
Illustration of a typical uni-modal (dashed) and two different bimodal molecular weight distributions (MWDs), reprinted with permission from [45]. Copyright 2012 Wiley.

Basically, all known polymerization technologies (slurry, gas phase, or solution) can be operated in a series of reactors in order to achieve multimodal PEs [36,37,38,39,40,41,42]. Examples are Hostalen (Lyondell-Basell) for the combination of slurry reactors and Unipol II (Dow) for the gas-phase technology. However, there are also combinations of different technologies such as Borstar (Borealis), which is an amalgamation of slurry and gas phases [43,44]. With all these technologies, bimodal molecular weight distributions (MWDs) can be produced, as illustrated in Figure 6 [45]. The vertical axis in this figure is the derivative of the cumulative weight fraction with respect to log Mw. The principal motivations for doing this are to improve performance in several regards, such as application properties (mechanical and rheological) [46,47,48,49], polymer morphology [39,50,51,52,53,54], and catalyst yield [54].

As an important material, PP has been widely used in many different fields including chemical, optical, and medical sectors [55,56,57,58]. The manufacture of PP is a billion-dollar business, which has seen about 5% annual growth rate in consumption in recent years. PP is synthesized using propylene in the presence of a catalyst and a co-catalyst (usually Al alkyls) at both laboratory and industrial scales [55,56,57,58,59]. Table 2 represents a historical timeline of the 20th century milestones in polyolefin production.

**Table 2:** Timeline showing the historical progress in the polymerization process of olefin—milestones are represented until the late 20th century.

Year	Progress in olefin polymerization process
1951	Hogan and Banks synthesizes crystalline polypropylene using chromium-NiO catalyst supported on silica alumina. (Subsequently, in 1983, the US patent office awards the patent to them for having substantial crystalline polypropylene content.)
1953	Karl Ziegler polymerizes ethene into high MW-HDPE (high density polyethylene) with the discovery of the catalyst based on titanium tetrachloride, and diethylaluminium chloride as co-catalyst.
1954	Giulio Natta, utilizes the catalyst suggested by Ziegler to produce PP. Ziegler and Natta are both awarded the Nobel Prize for Chemistry 1963 in recognition of their work on the Ziegler-Natta catalyst.
1957	Commercial production of PP commence in Italy, Germany, and USA. Natta and Breslow, independently discover metallocene catalyst to catalyze olefin polymerization with conventional co-catalyst (Al alkyls).
1961–1980	PP is used for manufacturing various products like fibers, fabrics, upholstery, nonwoven fabrics, and others on a commercial scale.
1973	2nd generation Ziegler Natta catalysts introduced with TiCl_3_ purple phases at lower temperatures.
1975–1978	3rd generation catalysts supported on MgCl_2_ commercialized by many companies.
1977–1980	Kaminsky and Sinn discover high activity metallocene single-site catalysts (SSCs) using methylaluminoxane (MAO) as co-catalyst.
1984	Ewen at the Exxon Company (USA) demonstrate that appropriate titanocenes render partially isotactic polypropylene.
1991	Fourth generation Ziegler Natta catalysts based on aluminium-oxane activated metallocene complexes used.
1995–1998	Brookhart and co-workers discover non-metallocene SSC based primarily on chelated late transition metals. Brintzinger and co-workers report on the synthesis of chiral bridged (“ansa”) metallocenes for homogeneous stereospecific 1-olefin polymerization [59]. Exxon Mobil and other companies commercialize PP using SSC.
1997	Montel (or Lyondell Basell) commercialize PP based on 5th generation Ziegler-Natta catalyst that use 1.3-diethers, and succinated as donors.

In coordination polymerization, generally a polyolefin is produced by multiple insertions of olefins into a metal-carbon bond in different ways. The regiochemistry of insertion (the catalyst regioselectivity and the regioregularity of the polymer) is determined by either primary or secondary olefin insertion into a metal-carbon bond, while the choice of the olefin enantioface selectivity determines the stereochemistry of each insertion (the catalyst stereoselectivity). The catalyst stereoselectivity (and the stereoregularity or tacticity of the polymer) is defined by the stereochemical relation between the stereogenic carbon atoms in the polymer chain, because any olefin insertion forms a new stereogenic center [60].

**Figure 7:**
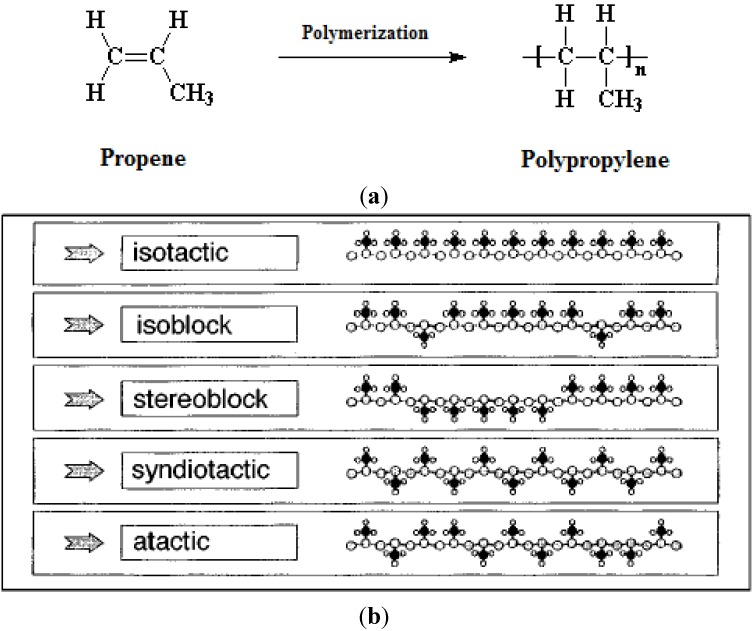
(**a**) Two-dimensional representation of linear PP that results from the arrangement of monomer units along the polymer chain during the polymerization process; and (**b**) portions of linear PP with different orientations of pendant methyl groups along the polymer backbone, reprinted with permissions from [58]. Copyright 2011 RSC.

Since propylene is an asymmetrical monomer, PP can be produced with different stereochemical configurations. Figure 7a,b shows the polymerization of propylene and PP’s different forms, *i.e.*, isotactic, hemi-isotactic, syndiotactic, and atactic [58,61,62,63,64,65]. The structure is based on the type of metal catalyst with tunable properties and selectivities [61,66,67,68,69]. From a commercial viewpoint, isotactic PP has a more ordered structure and therefore higher melting point, heats of fusion, and crystallinity in comparison to its atactic or syndiotactic forms.

The drawback when using Ziegler-Natta isotactic PPs lies in the fact that some structural parameters are almost impossible to study separately; for example molecular weight and tacticity are strongly coupled in these polymers [70]. In fact, isotactic Ziegler-Natta PPs can be considered as a mixture of very different types of chains: short atactic chains are present even in most isotactic commercial PPs [28]. Obtaining isotactic PP of varying molecular weight, while keeping isotacticity approximately constant, is not possible. Thus, evaluating the separated effect of molecular weight and tacticity and tacticity distribution appears almost impossible [70]. However, metallocene PPs are more homogeneous both in molecular weight, in tacticity, and tacticity distributions; chains resemble one another much more than when using Ziegler-Natta catalysts because of the presence of only one active center in metallocene catalysts [66].

While in metallocene PPs, the distribution of stereo defects is homogeneous, in their Ziegler-Natta counterparts the formation of stereo blocks takes place [70]. As a consequence of the homogeneous distribution of stereo defects, predominantly isotactic PPs synthesized using metallocene catalysts have shorter average isotactic sequences than Ziegler-Natta PPs with the same average of stereo defects; these possess no isotactoid or “atactic” blocks in their chains [28]. This structural difference is large enough to expect very different behavior from metallocene PPs as compared to Ziegler-Natta PPs in some properties. As a consequence of the different configurational structure, for the same tacticity, metallocene PPs show a lower melting point than Ziegler-Natta ones, and this difference is largely due to the lower isotacticity of the polymer [70]. In addition, large molecular weights are more difficult to obtain in metallocene polyolefins. Due to these features the applications of these newer polyolefins have been significant in the elastomeric, low tacticity types than in the high isotactic, high-melting point ones [71].

Out of several PP manufacturing processes, the gas-phase has acquired much interest as it is cost effective and also involves less consumption of raw materials and utilities [69]. However, catalysts are required to control the molecular weight of polymers, molecular weight distribution, copolymerization ratio, as well as the regio- and stereo-selectivities within the context of designer polymers. The development of such catalysts is a challenge to the polymer industry. To meet this challenge, clarification of the relationship between the structure of the active site and the catalyst performance on the basis of a precise and quantitative understanding of the polymerization mechanism of the catalyst α-TiCl_3_/Al(C_2_H_5_)_3_ is reported by Shiga [19].

**Figure 8:**
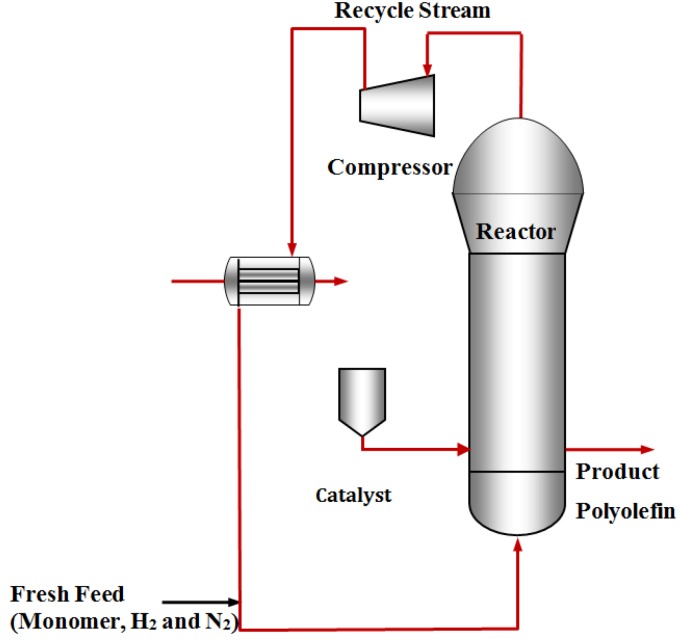
A process flow diagram representing the polymerization process of olefin in a gas-phase fluidized bed reactor.

A simple process flow diagram for the gas-phase olefin polymerization process is shown in Figure 8 [56]. The feed gas stream provides monomer, hydrogen, and nitrogen, and at the same time agitates and fluidizes the reactor bed (not shown in Figure 8 but a more detailed diagram of the reactor with various control loops is given elsewhere [69]) through the distributor and also removes the heat of the polymerization reaction. Polymerization occurs in the fluidized bed in the presence of Ziegler-Natta catalyst and triethyl aluminum co-catalyst. The unreacted gas exits the top of the reactor and is then compressed and cooled before being fed back into the bottom of the fluidized bed. The polymer production rate in this system is limited by heat removal from the circulating gas since the polymerization reaction is highly exothermic [69]. To maintain acceptable polymer production rate, which is an important goal for industry, it is necessary to keep the bed temperature above the dew point of the reactants to avoid gas condensation and below the melting point of the polymer to prevent particle melting, agglomeration, and consequent reactor shut down. For these reasons, process stabilization for propylene polymerization in a fluidized bed reactor is a challenging problem to be addressed through an efficient control system design.

In recent times, mathematical modeling and control of gas phase propylene polymerization have been reported in the literature to address the aforementioned issue [55,56,69]. Besides this, not much work, however, has been done on this topic until now due to many factors, such as the high non-linearity of the process dynamics involving complicated reaction mechanisms, complex flow characteristics of gas and solids, various heat and mass transfer mechanisms, and the interaction between the process control loops.

## 3. Role and Type of Catalysts

Since the discovery of PP, a wide variety of different catalysts have been designed and developed, leading to tailored polymers of entirely different structures, and applications by controlling polymer tacticity, molar mass, and molar mass distribution [72]. As defined earlier, a catalyst is used to reduce the activation energy for the polymerization process thereby speeding up the reaction and allowing it to proceed even under mild conditions. For instance, in the absence of the catalyst, ethylene does not undergo polymerization in mild conditions and requires high-energy particle collisions to react. Hence, the proportion of different structures formed is dependent on the relative rates of their formation [73].

In the PP industry, Ziegler-Natta catalysts play a vital role in production; however, to date the working mechanism of Ziegler-Natta systems have not been understood completely. An understanding of this behavior would help in designing and developing catalysts with desirable properties. Studies by Ronkko and co-workers [74] reveal that polymerization and fragmentation behavior of catalysts is dependent on the type of catalyst and nature of the catalyst support [75,76]. The catalyst should have (i) high porosity to allow good reactant diffusion; (ii) high mechanical strength to withstand thermal or chemical shocks while simultaneously possessing the ability to break up during polymerization; (iii) the ability to undergo fragmentation to yield desirable polymer content without having large contaminated fragments in the final product; and (iv) a decent distribution of active sites to ensure an even allotment of the final polymer product [75,76,77,78,79,80,81,82,83,84,85,86,87,88,89,90,91,92].

LLDPE could be produced using Ziegler-Natta catalysts that results in a blend of copolymers with each active site giving random distributions [17,36,93]. Metallocene catalysts, although result in random distributions, can sometimes provide regular co-monomer distributions, especially when the metallocene supramolecular structure enables tailoring of the macromolecular configuration [7,15,29,94,95,96].

Kaminsky showed that a co-catalyst system based on zirconocene (homogeneous Ziegler-Natta catalysts based on complexes of Zr, and used in combination with different organo-aluminum co-catalysts) and MAO is very active for the copolymerization of ethylene and oct-1-ene [94,97]. This zirconocene/MAO co-catalyst was used to prepare several ethylene-α-olefin copolymers in which oct-1-ene, dodec-1-ene, octadec-1-ene, and hexacos-1-ene were used as co-monomers. Obtained LLDPEs had regular side-chain distributions along the main chain and their properties were the subject of several studies [32,33,98,99].

**Scheme 1:**
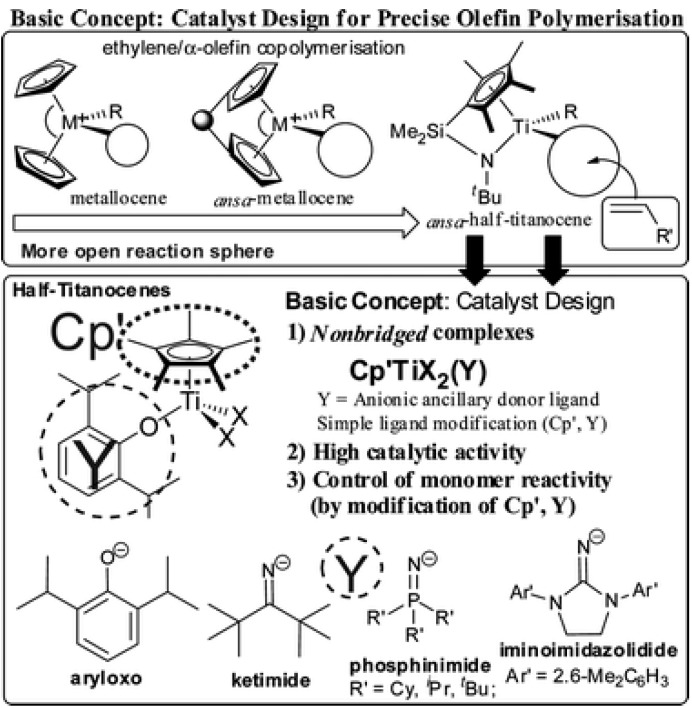
Basic proposed concept for the catalyst design and selected examples for half-titanocenes as effective catalyst precursors for olefin polymerization, reprinted with permission from [3]. Copyright 2013 World Scientific.

It has been reported that bridged (metallocene) type complexes show better co-monomer incorporation than the non-bridged (un-bridged) analogs in ethylene/α-olefin co-polymerization [100,101], although both steric and electronic factors affect the catalytic activity and molecular weight of resultant polymers in ethylene polymerization by means of substituted zirconocenes. The reason for this is that the bridged metallocenes possess a rather large coordination space compared to the non-bridged analogs, allowing better accessibility for the bulky α-olefins (Scheme 1) [100,101,102,103,104]. Linked half-titanocenes containing amide ligands, such as [Me_2_Si(C_5_Me_4_)(NtBu)]TiCl_2_[104], so called “constrained geometry catalysts (CGC)”, have also been known to exhibit efficient co-monomer incorporation (Scheme 1) [105,106,107,108,109,110]. Constrained geometry catalyst technology (CGCT) is based on homogeneous, single-site catalysts (SSCs) that allow for property design and optimization, and are capable of preparing homogeneous polyolefin copolymers [87]. The catalyst technology is based on a constrained geometry ligand attached to a transition-metal catalyst center. The strong Lewis acid systems are used to activate the catalyst, *i.e.*, to act as co-catalysts. The catalyst activity is based on Group 4 (IV) transition metals (e.g., titanium), which are covalently bonded to mono-cyclo-pentadienyl groups bridged with a hetero atom. As a result of bonding in the three components, a constrained cyclic structure is formed with the transition metal center. Besides tailoring of molecular structures, steps are taken to produce cost-effective efficient systems.

The efficiency of α-olefin in ethylene/α-olefin co-polymerization, that can be evaluated by using *r_E_* (reactivity ratio of ethylene) values under similar conditions, increases in the order: ZrCp_2_Cl_2_ < *rac*-Me_2_Si[benz(e)Ind]_2_ZrCl_2_ < [Me_2_Si(C_5_Me_4_)(NtBu)]Ti–Cl_2_ (where Cp = cyclopentadienyl, Me = methyl, tBu = *tert*-butyl, rac = racemic “diads” of chiral centers of the polymer, benz = benzene, ind = indenyl ligand) [103]. Further discussion on this topic is given in Section 3.3 and the reader is also referred to the survey written by Cano and Kunz [107] for more details.

Zirconocenes, as Kaminsky and others have shown, are 10–100 times more active than titanocenes and the classical Ziegler catalyst (activities are up to 875,000 kg PP mol^−1^·Zr^−1^·h^−1^) [7,111,112]. The activity of the former is also maintained at nearly the same level for several days. In addition, titanocenes cannot be used at higher temperatures and for longer polymerization times because the titanium (IV) is then reduced to the inactive titanium (III). Hafnocenes are about 10 times less active than titanocenes but produce PE with a higher molecular weight. Under the condition that every zirconocene complex forms a polymerization active site [113] the most active zirconocene produces about 15,000 polymer chains per hour at a polymerization temperature of 90 °C [112]. Further details on this, as well as activators, are given in Section 3.4.

The ansa zirconocene [En(THind)_2_]ZrCl_2_ exists in three structures as illustrated earlier by Kaminsky [112]. The rotation of the indenyl rings is hindered by the CH_2_–CH_2_–bridge. Beside the racemic mixture of the R and the S form, a meso form is possible. In the case of [En(THind)_2_]ZrCl_2_only traces of the meso form are obtained, which can be eliminated by recrystallization of the complexes. The meso form has no symmetry and produces therefore atactic PP similar to the un-bridged ZrCp_2_Cl_2_/MAO catalyst [61,62,114,115].

According to Shiga, the crystal structure of TiCl_3_ plays an important role in stereospecific polymerization of propylene [19]. Four crystalline modifications of TiCl_3_ have been reported [116]: α-, γ-, δ-forms (violet), and the β-form (brown). The layer structure of violet TiCl_3_ produces highly isotactic PP, whereas β-TiCl_3_ being fiber-shaped, gives a low yield of atactic PP. The mode of stacking of the common bi-dimensional TiCl_3_ sheets in layer structures leads to the difference in these three forms of violet TiCl_3_. The α-form of TiCl_3_ is specified by the layers that exhibit hexagonal close-packing of the chlorine atoms, whereas cubic close-packing has been found in γ-forms of TiCl_3_. However, in the case of δ-TiCl_3_, the mode of stacking of the structural layers is given by some statistical average of the modes of packing in the α- and γ-forms. The δ-form of TiCl_3_ is obtained by grinding α- or γ- TiCl_3_ [16,117]. Boor reported that δ-TiCl_3_ is used in the production of PP due to its high catalytic activity [118]. Keii reported on the effects of grinding α-TiCl_3_ on the polymerization of propylene [119]. The rate of propagation was proportional to the specific surface area of the TiCl_3_ under steady-state conditions, provided that the “true” specific surface area was evaluated by treating the TiCl_3_ with solvent in order to allow it to de-agglomerate.

### 3.1. Kinetic Study of Olefin Polymerization in General

Heterogeneous Phillips and Ziegler-Natta catalysts generally contain multiple types of active siteswhich results in the production of polymers having broad and, sometimes multimodal, microstructural distributions. Metallocene catalysts contain a single type of active site that is employed to produce polyethylene and polypropylene with entirely different microstructures from those produced by Ziegler-Natta and Phillips catalysts. Polyethylene and polypropylene produced by metallocene catalysts have uniform microstructures, with narrow molecular weight distribution and chemical composition distribution.

The general olefin polymerization (polyethylene and polypropylene) mechanisms that are acceptable for homopolymerization and copolymerzation by coordination polymerization with either Ziegler-Natta, Phillips or Metallocene catalysts are given below. Details of the kinetic model are reported by Soares [120].

(1) Elementary chemical reactions of olefin homopolymerization system.Initiation
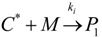
(1)Propagation
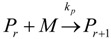
(2)Transfer 
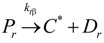
 β-hydride (3)

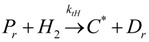
 to hydrogen (4)

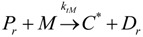
 to monomer (5)

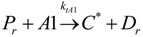
 to cocatalyst (6)Deactivation
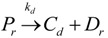
(7)

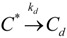
(8)Poisoning
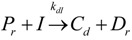
(9)(2) Elementary chemical reactions of olefin copolymerization system.Initiation
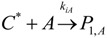
(10) 
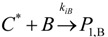
(11)Propagation 
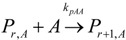
(12) 
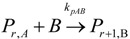
(13) 
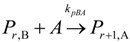
(14) 
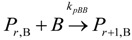
(15)Transfer 
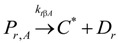
 β-hydride(16)

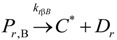
 β-hydride(17)

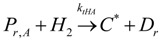
 to hydrogen(18)

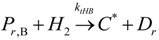
 to hydrogen(19)

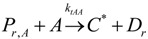
 to monomer(20)

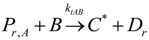
 to monomer(21)

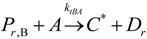
 to monomer(22)

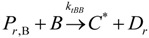
 to monomer(23)

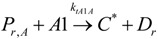
 to cocatalyst(24)

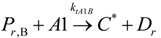
  to cocatalyst(25)Deactivation
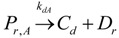
  Deactivation(26)

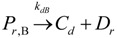
  Deactivation(27)

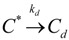
  Deactivation(28)Poisoning
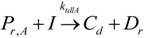
  Poisoning(29)

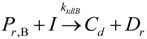
  Poisoning(30)

### 3.2. Electron Donors

Organic electron donors, such as esters, ethers, and alkoxysilanes, are widely used in catalyst preparation and polymerization processes, which play key roles in enhancing isotacticity and regulating molecular weight distribution of the PP products [121,122,123]. The electron donor added in the process of catalyst preparation is called the internal electron donor (Di), and the electron donor added in the polymerization process is called the external electron donor (De). In recent decades, the most commonly used catalyst in PP production contains phthalate as Di and alkoxysilane as De [123]. With such catalysts, PP with high isotacticity and controllable molecular weight can be produced at a very high catalytic efficiency [124].

Since the discovery of TiCl_4_/Di/MgCl_2_–AlR_3_/De type propylene polymerization catalysts in the early 1980s, great efforts have been paid to disclosing and understanding the mechanism of electron donor effects, with an aim of further improving the chain structure of PP by applying new Di/De combinations [125,126,127,128,129,130,131,132,133,134,135,136]. The main role of Di has been proposed to control the amount and spatial distribution of TiCl_4_ adsorbed on the MgCl_2_ crystallite surface [123]. When TiCl_4_/Di/MgCl_2_ type catalysts are treated with an AlR_3_/De mixture, most of Di molecules in the catalyst are quickly replaced by De, implying that the De plays more important roles in the polymerization system. The effects of De on stereoselectivity of active centers have been ascribed to reversible adsorption of donor on metal atoms (Mg or Ti) neighboring the central Ti metal of the active center. Busico *et al.* [137] have proposed a three-site model to explain the effects of De on catalyst efficiency and polymer stereoregularity. In this model, successive adsorption of De on the catalyst changes the stereochemical environment of the active center, turning aspecific centers into isospecific ones. A modified three-site model has been proposed by Liu and co-workers [138]. The mechanism of donor effects has also been studied based on investigation of the polymerization kinetics, including the effects of donor on the number and propagation rate constant of active centers [123]. Terano *et al.* [139] have investigated the effects of both Di and De on the number and propagation rate constants of different types of active centers based on stopped-flow polymerization experiments. By using a ^14^CO tagging method, Wang *et al.* [140] have compared the number and propagation rate constants of active centers of a series of catalysts containing different Di and De. According to this literature, addition of an external donor in the propylene polymerization system with MgCl_2_-supported Ziegler-Natta catalysts causes a decrease in the number of active centers ([C*]/[Ti]) and increase in the chain propagation rate constant (*k*_p_). These results suggest that deactivation of a part of active centers and properties alteration of the remaining active centers happen in parallel when De is added [123]. However, because the changes of the active center’s number and propagation rate constant with De/Ti molar ratio have not been experimentally determined, a detailed evaluation of the donor effects and quantitative comparisons between different external donors have seen limited investigations.

On the other hand, many theoretical studies on the mechanism of donor effects have been reported in the past ten years, using density functional theory (DFT) calculations as the main tool. Researchers, using DFT calculations, demonstrate that De molecules can coordinate on lateral cuts of MgCl_2_ crystallites in the catalyst [141,142,143,144,145,146]. Adsorption of the donor molecule on the adjacent positions of active sites increases their stereospecificity and changes their intrinsic activity. However, these conclusions are to be confirmed by more experimental evidence [123].

Fu and co-workers have developed a new method of counting active centers in propylene or ethylene polymerization with Ziegler-Natta catalysts, using 2-thiophenecarbonyl chloride (TPCC) as a quenching agent [147,148,149]. The method enables the determination of the number of active centers efficiently. Alkoxysilanes are widely used as De in industrial production of isotactic PP with TiCl_4_/Di/MgCl_2_ type Ziegler-Natta catalysts containing di-ester type Di. Previous studies show that the size of alkyl groups in alkoxysilane influences the catalyst activity, as well as the microstructure and the molecular weight characteristics of the PP product [127]. However, influence of De structure on the active center distribution is scarcely reported [123].

### 3.3. The Contribution of Metallocene-Related and Group 4 Ziegler-Natta Catalysts to the Advancement in Olefin Polymerization Processes

The strategy to develop metallocene-related catalysts has been put forth by high activity and tunable stereo- and regio-selectivity of metallocene-based olefin polymerization catalysts [59,64,150]. Sinn and co-workers introduced the activation of small amounts of water on the system Cp_2_MtX_2_/AlMe_3_ (X = Cl or alkyl group) and the subsequent controlled synthesis of MAO [151]. This provided organometallic and polymer chemists with a potent co-catalyst able to activate group 4 metallocenes (and a large number of other transition metal complexes, too) towards the polymerization of virtually any 1-olefins, as well as several cyclic olefins [65]. Over the past 30 years, these homogenous SSCs have dominated the literature due to a greater understanding of the mechanism of polymerization of ethylene leading to opportunities for designing and developing improved classes of catalysts [64,150,151,152,153,154,155,156,157,158,159,160,161,162,163,164,165,166,167,168,169,170,171,172,173,174,175,176,177,178]. However, the activity of Cp_2_-MtX_2_/MAO catalysts was moderate with propylene and, more importantly, did not produce stereo-regular polymers [65]. Very low molecular weight, atactic oils were obtained in all cases instead.

**Scheme 2:**
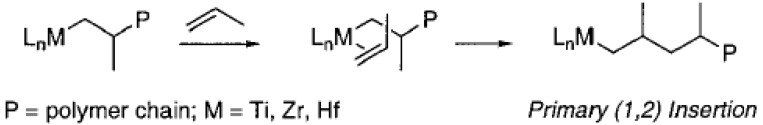
Insertion mechanism for olefin polymerization for Group 4 Ziegler-Natta catalysts, reprinted with permissions form [160], Copyright 2002 ACS.

Grubbs and Coates demonstrate the insertion mechanism for olefin polymerization for group 4 Ziegler-Natta catalysts [152], which occur by the coordination of an olefin to a vacant site followed by migratory insertion of the coordinated olefin into the growing polymer chain (Scheme 2) [153,154,155,156]. α-olefin insertions into metal-alkyl bonds occur predominately with primary (1,2) regio-chemistry both for Ziegler-Natta catalysts and metallocenes and the un-substituted alkene carbon becomes bound to the metal. The results obtained by Grubbs and Coates [152] are in agreement with theoretical observations [157,158,159]. Although, small amounts (<1%) of regio-errors are commonly observed in PPs synthesized using metallocene catalysts, especially with iso-specific zirconocenes [65,152], the lower catalytic activity and molecular weights of the polymers obtained through competing chain-release processes occur as a result of 2,1-insertions [161,162]. The rate of insertion is slow due to the higher barrier to olefin insertion into the more-bulky secondary metal-alkyl species, which are in competition with β-hydride elimination and chain-end isomerization (1,3-insertion); this increases with chain growth.

Hustad and co-workers have successfully proposed the secondary insertion of propylene in a group 4 catalyst system using bis(phenoxyimine)-based titanium catalysts [160]. In summary, the authors have discovered a highly unusual mechanism for the catalyst system that consists of both primary and secondary Ti-alkyl chains. The 2,1-insertion of propylene into a secondary titanium-alkyl (Ti-alkyl) is the dominant mechanism, whereas, insertions into the primary Ti-alkyl proceed with random regio-chemistry; end group analysis reveal that insertions into Ti-hydride are exclusively primary. Both heterogeneous titanium catalysts [69,163] and titanium-based metallocenes [67] produce ethylene-propylene copolymers (EPs) with high fractions of odd-numbered methylene sequences (*n* = 1, 3, 5) as a result of the highly regio-regular primary propylene insertion; resonances corresponding to the even-numbered sequences of length (two and four) are not observed (Ψ_2_ = Ψ_4_ ≈ 0). On the other hand, EPs synthesized using vanadium catalysts contain higher fractions of even-numbered methylene sequences (Ψ_2_ = 0.14, Ψ_4_ = 0.08) due to a high number of propylene inversions [164,165,166,167,168,169].

**Scheme 3:**
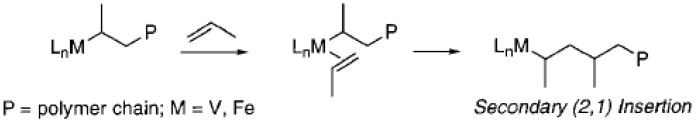
Polymerization of α-olefins by a secondary insertion mechanism, reprinted with permissions form [160], Copyright 2002 ACS.

It has been observed that with the exception of Group 4, catalysts based on other metals such as vanadium [170,171], nickel [172,173], palladium [173], and iron [174,175] have also been reported to polymerize α-olefins by a secondary insertion mechanism (Scheme 3). Wu and Li [171], as well as Pellecchia and co-workers [172], have reported syndiotactic-specific polymerization with vanadium and nickel, respectively, while other workers have discussed about isotactic PP from iron-based catalysts [174,175,176].

**Scheme 4:**
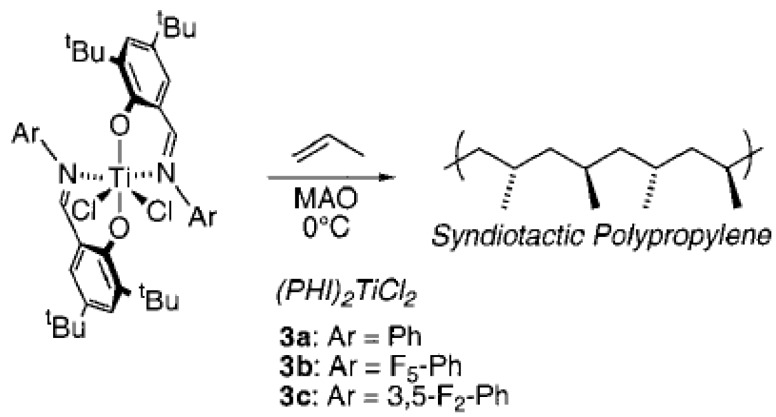
Stereo-selective α-olefin polymerization of propylene, reprinted with permission form [160], Copyright 1996 ACS.

**Scheme 5:**
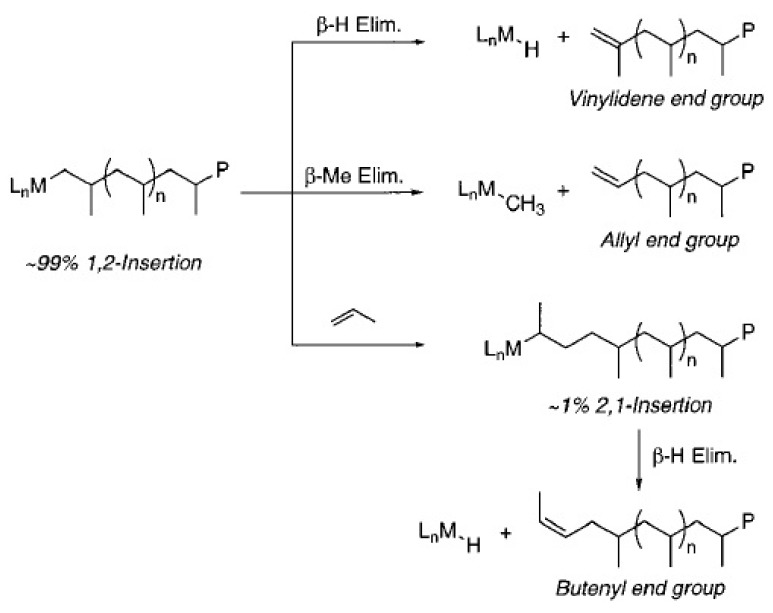
Metallocenes that yield PPs with characteristic end groups resulting from chain-release reactions, reprinted with permission form [160], Copyright 2002 ACS.

Hustad and co-workers [160] discovered a new family of catalysts while exploring the non-metallocene species for stereo-selective α-olefin polymerization of propylene (Scheme 4) [178,179]. The same authors reported upon the formation of complexes that were capable of catalyzing the highly syndio-specific and controlled polymerization of propylene as well as synthesizing ethylene and propylene-based block copolymers [180]. Scheme 5 refers to the metallocenes that gave PPs with characteristic end groups resulting from chain-release reactions, primarily β-hydride and/or β-methyl transfer [161]. In processes that competed with chain growth, these catalysts participated in different types of chain release, generating a free polymer chain along with an active metal-hydride or metal-alkyl species capable of propagating a new polymer chain [160].

**Scheme 6:**
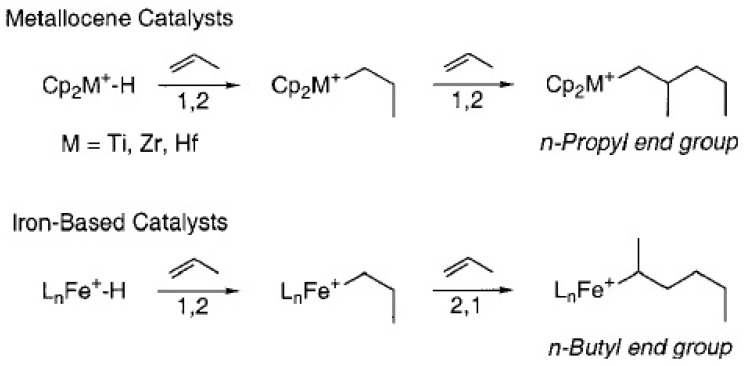
n-butyl initiated PP formation due to an unusual secondary insertion mechanism, reprinted with permission form [65,160], Copyright 2002 ACS and 2013 RSC.

**Scheme 7:**
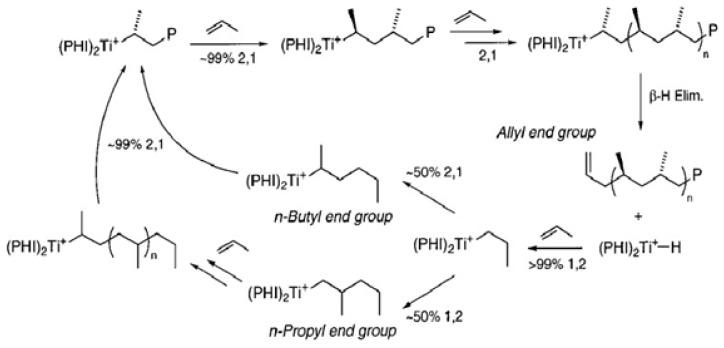
The initiation, propagation, and chain release reactions for propylene polymerization by the phenoxyimine-based catalyst system, reprinted with permission form [160], Copyright 2002 ACS.

The metal-hydride or metal-methyl species produced by chain release in olefin polymerizations are capable of growing a new polymer chain. For metallocene catalysts, primary insertions into metal-hydrides produce n-propyl initiated PP (Scheme 6) [65], whereas, those inserted into the metal-methyl species are responsible for isobutyl end groups. The PPs produced from the iron-based catalyst contain saturated end groups; this generates n-butyl initiated PP due to the unusual secondary insertion mechanism (Scheme 6) [180]. Scheme 7 represents the initiation, propagation, and chain release for propylene polymerization by the phenoxyimine-based catalyst system. The primary insertion into Ti-hydride initiates the reaction producing n-propyl species. However, chain propagation reactions of propylene on the Ti-alkyl species can be considered as four distinct processes: (a) primary insertion into a primary metal-alkyl; (b) secondary insertion into a primary metal-alkyl; (c) primary insertion into a secondary metal-alkyl; and (d) secondary insertion into a secondary metal-alkyl species. The processes represented in (a) and (b) are important only in the initial stages of polymerization and occur in almost equal proportions, while process (c) accounts for *ca.* 1% of total propylene enchainment. Despite the specific nature of insertion into the primary Ti-alkyl species, statistics reveal that a secondary titanium-alkyl compound could be generated, after which propagation becomes exclusively secondary as for process (d). The chain release then occurs exclusively by β-hydride transfer from the terminal methyl species, giving PP with allylic end groups [65,160]. It has been observed that metallocenes do not undergo this type of chain transfer, whereas, they act as the major source of termination when applied with iron-based catalysts.

Yu and co-workers [85] have recently investigated the chain-transfer reactions of TiCl_4_/MgCl_2_–AlEt_3_ catalyzed propylene polymerization under conditions of severely starved monomer supply with suppressed chain transfer to the co-catalyst system. Besides 1-propen-2-yl (vinylidene) end group formation by β-H transfer after primary (1,2-) insertion, 1-propen-3-yl (allyl) end group formation by β-Me (β-methyl) transfer after 1,2-insertion and 2-buten-4-yl formation by β-H transfer after secondary (2,1-) insertion were also detected in the polymeric product by ^1^H-NMR analysis [181]. The monomer dependencies of the chain-transfer reactions were also studied [182]. On account of the uni-molecular nature of β-H transfer after a secondary insertion procedure, the content of 2-buten-4-yl end group, which is too low to be detected during PP formation under conventional conditions, was found to increase markedly in the product of the monomer-starved polymerization process [85].

### 3.4. Olefin Polymerization by Half-Titanocenes Containing Aryloxy Ligands

It has long been known that the ligand modification is very important in order for metal catalyzed olefin polymerization to proceed with remarkable activities [183,184]. For example, as shown in Table 3 and Table 4, both substituents on cyclopentadienyl (Cp' and the * denotes a radical) and aryloxo ligands affect the activity for ethylene polymerization [185,186,187,188]. Cp*TiCl_2_(O-2,6-*^i^*Pr_2_C_6_H_3_), where *^i^*Pr represents iso-propylene, exhibits notable activities, and the activity by Cp'TiCl_2_(O-2,6-*^i^*Pr_2_C_6_H_3_) increases in the order: Cp' = Cp* ≫ 1,3-tBu_2_C_5_H_3_ > 1,3-Me_2_C_5_H_3_, tBuC_5_H_4_≫ Cp (Table 3).

**Table 3:** Effect of cyclopentadienyl fragment (Cp') on the activity of olefin polymerization by Cp'TiCl_2_(O-2,6-*^i^*Pr_2_C_6_H_3_)–MAO catalyst systems ^a^ [186]. Copyright 2011 RSC.

Cp' (μmol)	Olefin	Activity ^b^	TON ^c^	10^−4^*M*_w_^d^	*M*_w_/*M*_n_^d^
Cp (18.3)	Ethylene	77	2,750	–	–
Cp (5.0)	1-Hexene	62	370	0.68	–
*^t^*BuC_5_H_4_ (15.1)	Ethylene	258	9,200	5.99	2.1
*^t^*BuC_5_H_4_ (5.0)	1-Hexene	90	532	8.04	1.6
*^t^*BuC_5_H_4_ (5.0)	1-Octene	125	558	8.25	1.9
1,3-Me_2_C_5_H_2_ (24.2)	Ethylene	215	7,660	1.75	2.5
1,3-Me_2_C_5_H_2_ (5.0)	1-Hexene	184	1,090	8.73	1.9
1,3-*^t^*Bu_2_C_5_H_2_ (5.0)	Ethylene	653	23,300	64.9	6.8
1,3-*^t^*Bu_2_C_5_H_2_ (5.0)	1-Hexene	26	152	2.16	1.6
1,3-*^t^*Bu_2_C_5_H_2_ (5.0)	1-octene	38	168	1.75	1.5
Cp* (6.5)	Ethylene	2,220	79,100	45.9	5.0
Cp* (1.0)	1-Hexene	728	4,330	69.4	1.6
Cp* (1.0)	1-Octene	970	4,320	49.5	1.8
Cp* (1.0)	1-Decene	1036	3,690	41.7	1.7

^a^ Cited from [185,187]. Conditions: ethylene 4 atm., 60 °C, 1 h, toluene 300 mL, [Ph_3_C][B(C_6_F_5_)_4_]/Al*^i^*Bu_3_/Ti = 1/500/1 (molar ratio); α-olefin 5 mL, catalyst 2 μmol·mL^−1^ toluene, MAO white solid, 25 °C, 30 min. ^b^ Activity in kg-polymer mol^−1^·Ti^−1^·h^−1^. ^c^ TON (turnover numbers) = molar amount of olefin reacted per mol-Ti. ^d^ By GPC *vs.* polystyrene standards.

**Table 4:** Effect of aryloxo substituents toward the activity of ethylene polymerization by Cp*TiCl_2_(O-2-R^1^-4-R^2^-6-R^3^-C_6_H_2_)–MAO catalyst systems ^a^.

R^1^, R^2^, R^3^ (μmol)	Activity ^b^	TON ^c^	10^−4^*M*_w_^d^	*M*_w_/*M*_n_^d^
*^i^*Pr, H, *^i^*Pr (4.2)	1,240	43,100	64.9	4.7
H, Me, H (13.0)	25	890	–	–
Me, H, Me (4.0)	1,000	35,700	123	4.5
*^t^*Bu, Me, Me (13.0)	446	15,900	–	–
Me, Me, Me (8.4)	369	13,200	–	–

^a^ Cited from [186]. Conditions: ethylene 4 atm., 60 °C, 1 h, toluene 300 mL, MAO (Al/Ti = 1000, molar ratio). ^b^ Activity in kg-polymer mol^−1^·Ti^−1^·h^−1^. ^c^ TON (turnover numbers) = molar amount of reacted olefin per mol-Ti. ^d^ By GPC *vs.* polystyrene standards.

Similar results have been observed by several workers [188,189,190] for syndiospecific polymerization of styrene using a series of Cp'Ti(OMe)_3_ complexes. They explain the high activity by assuming that electron-donating substituents stabilize the active sites [189,190]. However, the tBu_2_Cp analog shows lower catalytic activity in 1-hexene and 1-octene polymerization due to the presence of steric bulk on Cp' [191]. Researchers have also monitored comparable levels of activities between 1-hexene and 1-octene polymerization reactions [192].

A series of half-titanocenes containing phosphinimide ligands of type, Cp'Ti(N=PR_3_)X_2_, were employed to explore the effect of the substituents on both Cp' and N=PR_3_ groups for activity during ethylene polymerization (Table 5) [186,193]. These complexes exhibited remarkable catalytic activities in the presence of MAO, which improved when they were used in combination with [Ph_3_C][B(C_6_F_5_)_4_]. Substituents on both Cp' and N=PR_3_ ligands played an essential role during the process and the use of the N=PCy_3_ ligand was effective.

The tBuC_5_H_4_ analogs were more suitable than the Cp analogs, suggesting that electron-donating substituents on Cp' increased the activity [186]. Analogous zirconium complexes were also prepared, but these complexes showed low activities for ethylene polymerization in the presence of MAO [194]; the activities by the Zr analogs improved in the presence of [Ph_3_C][B(C_6_F_5_)_4_] co-catalysts [195].

DFT (density functional theory) calculations on the polymerization mechanism by a series of catalyst models derived from CpTiMe_2_(N=PR_3_) (R = Me, NH_2_, H, Cl, F) were carried out by Beddie and co-workers [196]. The authors demonstrated the critical role of ion pairing in determining the overall barrier to polymerization and suggested that ligands containing electron-donating substituents could reduce this barrier. The tris-amido-phosphinimide analogs, Cp'TiX_2_[N=P(NR_2_)_3_] (X = Cl, Me), showed notable catalytic activities in the presence of borate-based activators (Table 6) and their activity improved upon increasing the steric bulk [197]. Optimization of steric bulk and electronic characteristics to facilitate ion-pair separation and prolonged catalyst lifetime were, thus, achieved, affording a readily accessible and easily varied family of highly active catalysts [196,197,198].

**Table 5:** Ethylene polymerization by Cp'TiX_2_(N=PR_3_) [**4**, Cp' = Cp, ^*t*^BuC_5_H_4_ (^*t*^BuCp); X = Cl, Me; R = Cy, ^*i*^Pr, ^*t*^Bu]–co-catalyst systems ^a^, reprinted with permissions form [186,193]. Copyright 2011 2013 RSC.

Complex	Co-Catalyst	Activity ^b^	10^−4^*M*w ^c^	*M*w/*M*n ^c^
CpTiCl_2_(N=PCy_3_)	MAO	42	0.36 ^*d*^	1.8
			33.6	2.2
CpTiCl_2_(N=P*^i^*Pr3)	MAO	49	1.87 ^*d*^	2.8
			57.9	2.4
CpTiCl_2_(N=P*^t^*Bu3)	MAO	500	8.99	2.4
CpTiMe_2_(N=PCy_3_)	Ph_3_CB(C_6_F_5_)_4_	231	13.5	2.8
CpTiMe_2_(N=P*^i^*Pr3)	Ph_3_CB(C_6_F_5_)_4_	225	16.4	3.4
CpTiMe_2_(N=P*^t^*Bu3)	Ph_3_CB(C_6_F_5_)_4_	401	16.6	3.4
*^t^*BuCpTiCl_2_(N=PCy_3_)	MAO	46	0.74 ^*d*^	2.1
			89.4	3.4
*^t^*BuCpTiCl_2_(N=P*^i^*Pr3)	MAO	16	0.76 ^*d*^	1.9
			91	2.5
*^t^*BuCpTiCl_2_(N=P*^t^*Bu3)	MAO	881	6.54	2.4
*^t^*BuCpTiMe_2_(N=PCy_3_)	Ph_3_CB(C_6_F_5_)_4_	1807	31	7.5
*^t^*BuCpTiMe_2_(N=P*^i^*Pr3)	Ph_3_CB(C_6_F_5_)_4_	1193	25.9	9.9
*^t^*BuCpTiMe_2_(N=P*^t^*Bu3)	Ph_3_CB(C_6_F_5_)_4_	1296	32.1	12.3
[Me_2_Si(C_5_Me_4_)(N*^t^*Bu)]TiCl_2_	MAO	630	–	–

^a^ Cited from [191]. Conditions: catalyst 0.01–0.03 mmol, toluene, ethylene 1 atm., 25 °C, 0.5–3 min, MAO (Al/Ti = 500, molar ratio) or Ph_3_CB(C_6_F_5_)_4_ (B/Ti = 2). ^b^ Activity in kg-polymer per mol-Ti·h^−1^. ^c^ GPC data *vs.* polyethylene standards. ^d^ Bimodal molecular weight distributions.

**Table 6:** Ethylene polymerization by Cp'TiX_2_[NP(NR^1^R^2^)_3_] (**5**)–co-catalyst systems ^a^, reprinted with permission form [186]. Copyright 2011 RSC.

Pre-catalyst (μmol·L^−1^)	Co-Catalyst	*t*/min	Activity ^b^	10^−4^*M*_n_^c^	*M*_w_/*M*_n_^c^
Cp*TiCl_2_[N=P(NMe_2_)_3_] (100)	MAO	30	21	82.6	1.72
Cp*TiCl_2_[N=P(NEt_2_)_3_] (100)	MAO	30	39	9.01	1.65
Cp*TiCl_2_[N=P{N(Me)*^i^*Pr}_3_] (50)	MAO	30	56	12.78	2.76
Cp*TiCl_2_[N=P{N(Et)Ph}_3_] (50)	MAO	30	200	12.61	4.02
CpTiMe_2_[N=P(NMe_2_)_3_] (4)	Al/B ^d^	10	2,200	31.5	2.05
CpTiMe_2_[N=P(NEt_2_)_3_] (4)	Al/B ^d^	10	3,500	39.4	1.91
CpTiMe_2_[N=P(NPr_2_)_3_] (4)	Al/B ^d^	10	5,500	–	–
CpTiMe_2_[N=P(NBu_2_)_3_] (4)	Al/B ^d^	10	3,600	–	–
CpTiMe_2_[N=P{N(Me)*^i^*Pr}_3_] (4)	Al/B ^d^	10	3,600	38.86	1.85
CpTiMe_2_[N=P{N(Et)Ph}_3_] (4)	Al/B ^d^	10	4,200	43.25	1.92
Cp*TiMe_2_[N=P(NMe_2_)_3_] (4)	Al/B ^d^	10	4,200	14.08	4.92
Cp*TiMe_2_[N=P(NEt_2_)_3_] (4)	Al/B ^d^	10	4,700	–	–
Cp*TiMe_2_[N=P(NPr_2_)_3_] (4)	Al/B ^d^	10	10,000	–	–
Cp*TiMe_2_[N=P(NBu_2_)_3_] (4)	Al/B ^d^	10	6,100	–	–
Cp*TiMe_2_[N=P{N(Me)*^i^*Pr}_3_] (4)	Al/B ^d^	10	4,900	28.81	2.14
Cp*TiMe_2_[N=P{N(Et)Ph}_3_] (4)	Al/B ^d^	10	4,200	32.46	2.03
Cp*TiMe_2_[N=P*^i^*Pr_3_] (4)	Al/B ^d^	10	5,200	49.34	2.05
CpTiMe_2_[N=P*^t^*Bu_3_] (4)	Al/B ^d^	10	5,600	43.78	1.8
Cp_2_ZrMe_2_ (4)	Al/B ^d^	10	16,000	17.5	1.89

^a^ Cited from [192]. *Conditions*: ethylene 2 atm. at 30 °C, toluene 600 mL, stir rate = 1000 rpm, 500 equivalent of MAO or Al/B. ^b^ Activity in kg-PE mol^−1^·Ti^−1^·h^−1^·atm^−1^. ^c^ GPC data in *o*-dichlorobenzene. ^d^ Al/B = Al*^i^*Bu_3_/B(C_6_F_5_)_3_, 2 equiv. of B(C_6_F_5_)_3_; 20 equiv of Al*^i^*Bu_3_.

## 4. Conclusions and Future Perspective

In this paper we have given a review of the changes and current state of PE and PP manufacturing processes, including role and types of catalysts and co-catalysts employed over the years. Even though Ziegler-Natta catalysts have been used significantly since their discovery, metallocene catalysts and co-catalyst systems have tended to replace them in recent times. We have reported upon the yields and mechanisms for the production of both PP and PE and have also provided a perspective on future research directions. More laboratory-scale work is recommended to understand the complexity of the polymerization process such that a greater amount of information is obtained for optimization purposes.

**Figure 9:**
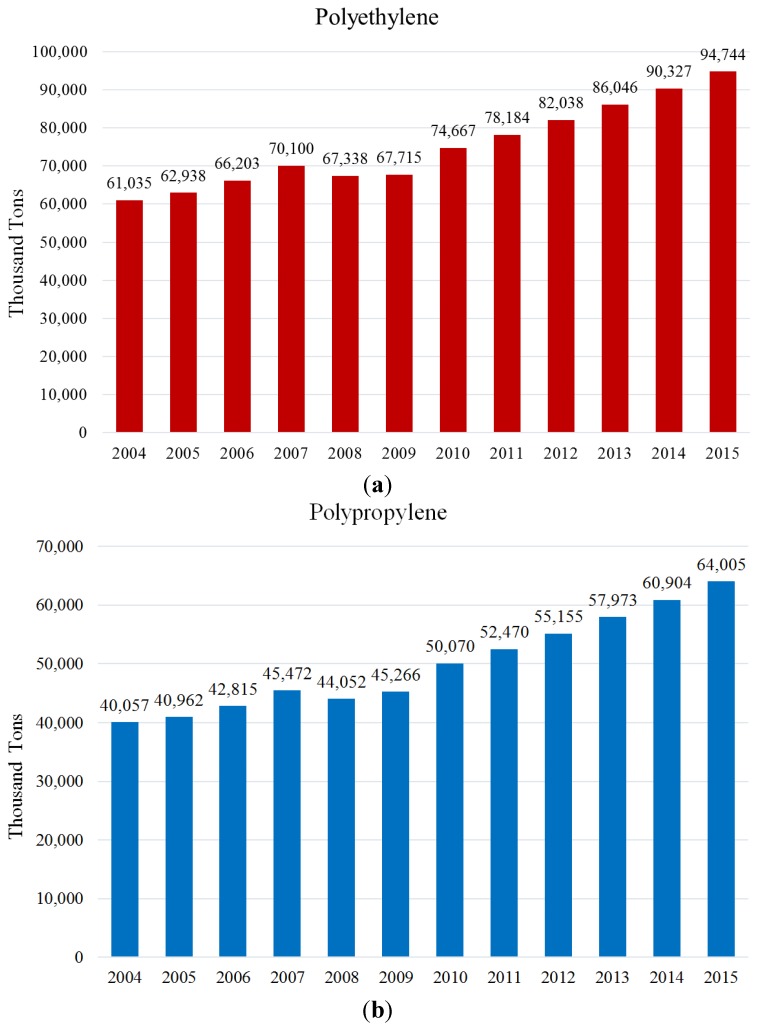
Bar chart representation of the world’s demand for PE (**a**); and PP (**b**) from 2004 to 2015, reprinted with permission from [199]. Copyright 2011 Canadian Plastics.

From the early 1990s, the polymer industry has been undergoing a strong revolution with the discovery of SSCs. These catalysts have paved the way to synthesizing tailored polymers with desired characteristics. Figure 9 illustrates the significant increase in the demand for PE and PP from 2004 to 2015 [199]. However, the industry has to be more cautious so as to maintain effective consumption of raw materials and utilities for PE and PP productions. The future of PE and PP is focused on the chemical resources available for polyolefins, well-designed copolymerization processes, miscellaneous composite types for the polymer with novel effective compatible compounds, as well as a full LCA (life cycle analysis) of the PE and PP products.

A recent perspective paper on PEs described many reports concerning syntheses of half-titanocenes and their potentials as ethylene polymerization catalysts [187]. We have included some useful tables from that work to highlight the significant contribution made to the scientific community by the authors. However, in most cases researchers have stopped their evaluations, focusing mainly on ethylene polymerization. More information is required concerning the catalytic stability in the reaction mixture, as well as on the electronic and steric effects toward both the activity and the co-monomer incorporation during the polymerization reaction. Complexes of such type may be effective for synthesis of new polyolefins by incorporation of monomers that are not successful as ordinary catalysts; these studies should, thus, be explored in the near future [187].

There are still many possibilities for the synthesis and processing of new types of polyolefin copolymers, especially for polymer blends with other polymers such as polyamides, polyesters, and polynitriles. For these blends polar co-monomers have to be incorporated into the PE or PP chain and more efficient catalysts have to be developed. Other new properties of polyolefins can be reached by means of block copolymers. A combination of single site catalysts is able to form, in a first step, a hard PP block, and in a second step, a soft ethylene/propylene (EP) copolymer. The first success in this field is described in the literature [200]. Similar block copolymers can be obtained by controlled polymerization of olefins [201,202]. For example, di-block copolymers of poly[syndiotactic propylene-b-(ethylene-co-propylene)] have been synthesized with a controlled molecular weight and a narrow molecular weight distribution (*M*_w_/*M*_n_ ≈ 1.1) [179]. Later on, bis(phenoxyimine)-titanium dichloride/MAO catalysts were used for the polymerization of 1,5-hexadiene to give a random copolymer with 1,3-methylenecyclopentane (MCP) and 3-vinyltetramethylene (VTM) units [203]. The VTM units in the copolymer have been shown to undergo a cross-metathesis reaction with alkenes catalyzed by a ruthenium carbene for additional functionalization of the copolymer [204].

Polyolefin nanocomposites will open up the approach to new classes of materials with special property combinations. The soft polyolefin matrix can be combined with hard inorganic particles, silicate layers, carbon nano fibers, or with carbon nanotubes, with extremely high tensile strengths. A possible means for the preparation of such polyolefin nanocomposites involves the *in situ* polymerization by metallocene catalysts [205,206]. Late transition metal complexes, which are more stable in water and ionic liquid solvents, can be used for emulsion polymerization, opening new fields of applications for polyolefins [207,208,209,210,211,212,213,214].

More active catalysts and adapted processes have to be developed. These efforts will pave the way for new promising possibilities for the evolution of new fine polyolefins with unique properties by incorporation of new co-monomers and/or by adopting new synthetic strategies. Considering the fact that the area is open to many directions, studies, and development, it would be heartening to observe novel discoveries being reported in the literature by academicians instead of confining majority of the research results within industries [56,57,215,216,217,218,219]. In addition, it may be worthwhile to synthesize polyolefin composites, which contain bio-degradable components that can be used for packaging materials in order to reduce their environmental impacts.

## Nomenclature

C*: active site
C_d_: deactivated site
D_r_: dead polymer of chain length r
H_2_ hydrogen: the most common chain transfer agent for these systems, except for Phillips catalysts
M: monomer
Al: cocatalyst
I: impurities
P_r_: living polymer chain of length r
